# Serial Increases in Human Leukocyte Antigen-DR Expression and Decreases in Interleukin-10 Expression in Alveolar Monocytes of Survivors of Pneumonia-Related Acute Respiratory Distress Syndrome

**DOI:** 10.3390/biology11121793

**Published:** 2022-12-09

**Authors:** Chien-Ming Chu, Chia-Jung Chung, Chih-Yu Huang, Chung-Chieh Yu, Chao-Hung Wang, Li-Fu Li, Huang-Pin Wu

**Affiliations:** 1 Division of Pulmonary, Critical Care and Sleep Medicine, Chang Gung Memorial Hospital, Keelung 20401, Taiwan; 2 College of Medicine, Chang Gung University, Taoyuan 33302, Taiwan; 3 Heart Failure Research Center, Division of Cardiology, Chang Gung Memorial Hospital, Keelung 20401, Taiwan

**Keywords:** HLA-DR, monocytes, bronchoalveolar lavage, acute respiratory distress syndrome

## Abstract

**Simple Summary:**

Patients with acute respiratory distress syndrome (ARDS) have high mortality. It is important to understand the complex immune interactions in ARDS, which may help identify potential therapeutic targets. In this study, we tried to determine the trends of human leukocyte antigen (HLA)-DR and cytokine expression in alveolar monocytes in patients with pneumonia-related ARDS. We found that alveolar monocyte HLA-DR expression (mHLA-DR) in survivors increased remarkably after seven days, and alveolar monocyte IL-10 expression in survivors decreased substantially after seven days. These findings highlighted the importance of serial increases in HLA-DR expression and decreases in IL-10 expression in alveolar monocytes of survivors.

**Abstract:**

ARDS is a potentially lethal syndrome. HLA-DR expression in monocytes reflects their activation and antigen-presenting capacity. However, the correlation between clinical outcomes and HLA-DR expression in alveolar monocytes/macrophages in patients with pneumonia-related ARDS remains unclear. Thus, we determined the trends of HLA-DR and cytokine expressions in alveolar monocytes using repeated measurements to answer this question. Thirty-one pneumonia patients with respiratory failure and ARDS without coronavirus disease 2019 between November 2019 and November 2021 were enrolled in our intensive care unit and three without complete data were excluded. Interleukin (IL)-10, IL-12, and HLA-DR expression in bronchoalveolar lavage (BAL) monocytes were determined on days one and eight. Monocyte HLA-DR expression (mHLA-DR) and CD4 T lymphocytes percentages in BAL cells of survivors increased remarkably after seven days. Monocyte IL-10 expression and monocytes percentages in BAL cells of survivors decreased substantially after seven days. The mHLA-DR was negatively correlated with disease severity scores on day one and eight. In conclusion, serial increases in HLA-DR expression and decreases in IL-10 expression were observed in BAL monocytes of survivors of pneumonia-related ARDS. More studies are needed to confirm this point of view, and then development of a therapeutic agent restoring mHLA-DR and preventing IL-10 production can be considered.

## 1. Introduction

Acute respiratory distress syndrome (ARDS) is a relatively common and potentially lethal syndrome with high mortality, ranging from 27% to 45% [1,2]. The most common risk factor for developing ARDS is pneumonia; unfortunately, no pharmacological therapy has been shown to reduce its mortality rate [3]. Thus, it is important to understand the complex immune interactions in the infected areas of patients with pneumonia-related ARDS, which may help identify potential therapeutic targets.

Human leukocyte antigen (HLA)-DR is a major histocompatibility complex class II molecule mostly expressed on monocytes/macrophages. The expression of HLA-DR usually represents the activation of immune cell and the capacity of antigen-presenting. There are abundant data showing that not only hyper-inflammation but also anti-inflammation is observed in patients with severe infection. Monocyte HLA-DR expression (mHLA-DR) controls the interplay between innate and adaptive immunity. It may also serve as an overall biomarker of immunosuppression [4]. More than a single value at a time point, a serial change in mHLA-DR over time could be a better predictor of mortality in patients with sepsis [5,6]. In addition to mHLA-DR, many cytokines are involved in the interplay between inflammation and anti-inflammation. The key cytokine involved in anti-inflammation and increased mortality is interleukin (IL)-10 [5,7]. IL-10 may promote ARDS development via inhibiting cell differentiation from stem cells to alveolar type II epithelial cells [8]. Jin et al. ever reported that IL-10-1082 G/G genotype was associated with lower development and mortality of ARDS in Chineses [9]. In an early study, mHLA-DR was a predictive marker for survival in sepsis, while serum IL-10 levels on days three and ten had negative prognostic value [10].

Patients with sepsis or ARDS have lower blood mHLA-DR compared to healthy controls [5,11]. Although blood is the most common sample used for research, bronchoalveolar lavage (BAL) fluid is the closest sample to the infection site of pneumonia and may accurately reflect the local lung environment. However, the correlation between clinical outcomes and HLA-DR expression in alveolar monocytes/macrophages of patients with pneumonia-related ARDS remains unclear. Bendib et al. found no strong association between alveolar mHLA-DR and hospital mortality in patients with pneumonia-related ARDS [11]. Unfortunately, they did not serially detect the HLA-DR expression in alveolar monocytes. The trend in HLA-DR expression in alveolar monocytes may be associated with mortality.

To better understand the pathogenesis of local infection sites in patients with pneumonia-related ARDS, we designed this study to determine the trends of HLA-DR and cytokine expressions in alveolar monocytes using repeated measurements.

## 2. Materials and Methods

### 2.1. Participants and Definitions

From November 2019 to November 2021, 31 patients reporting severe pneumonia with respiratory failure and ARDS without coronavirus disease 2019 admitted to our intensive care unit (ICU) were enrolled into this study. One patient was excluded due to lack of sufficient BAL fluid cells, and two patients were excluded due to unavailability of flow cytometry data. Informed consent was obtained from a close family member of the patient. The Institutional Review Board at Chang Gung Memorial Hospital approved this study (201601731A3, 201601731A3C501, 201801835A3). Survivors were defined as patients who were alive for ≥28 days after admission to our ICU. All comorbidities and histories of the subjects were recorded.

ARDS was defined according to the Berlin definition [2]. Pneumonia was defined as a new abnormal infiltration/opacity on chest radiography with respiratory symptoms or fever. Sepsis and septic shock were defined according to the Sepsis-3 guidelines [12]. Sepsis was a suspected or documented infection with an acute increase (≥2 points) in the sequential organ failure assessment (SOFA) score. Septic shock was sepsis with a blood lactate level >18 mg/dL and low blood pressure which was unresponsive to fluid resuscitation, requiring vasopressors to maintain a mean arterial pressure of ≥65 mmHg during the first three days after ICU admission. Respiratory failure was a ventilatory dysfunction requiring invasive ventilator support. Jaundice was defined as hyperbilirubinemia (total bilirubin >2 mg/dL), whereas thrombocytopenia was a platelet count <150,000/μL. Acute kidney injury was defined according to the stage 1, 2, or 3 of Kidney Disease Improving Global Outcomes guidelines [13]. Disease severity was evaluated based on the Acute Physiology and Chronic Health Evaluation (APACHE) II score [14].

Based on the condition of each patient, standard managements were provided to all patients, including initial fluid resuscitation, antibiotics use, vasopressor to maintain blood pressure, renal replacement therapy for acute renal failure, and low tidal ventilation, according to the guidelines [15].

### 2.2. BAL Mononuclear Cell Preparation

BAL mononuclear cell preparation was performed in a lung segment showing consolidation and infiltration on chest X-ray within 48 h of admission to the ICU (at 08:30 a.m.). For BAL fluid, the first aliquot of sterile saline (60 mL) was instilled via a bronchoscope wedged into the segmental bronchus. The instilled saline was gently retrieved using a negative suction pressure of <100 mmHg. Negative suction pressure should be adjusted to avoid visible airway collapse. The following two aliquots of sterile saline (60 mL) were prepared in the same manner as the first aliquot. To remove debris, the recovered volume was filtered through cotton gauze and stored in heparin tubes (5000 U/50 mL recovered volume). The day of the first BAL was defined as Day 1. Additional BAL was performed for workup on day eight unless the patient died, or was discharged from the ICU. BAL mononuclear cells were isolated via differential centrifugation over Ficoll-Plaque (Amersham Biosciences, Uppsala, Sweden) within 2 h of collection.

### 2.3. Flow Cytometric Analysis of BAL Mononuclear Cells

**Figure 1:**
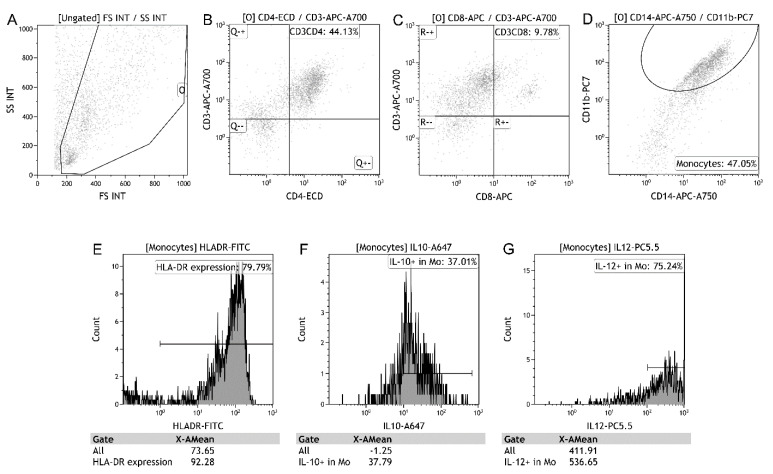
Flow cytometry of bronchoalveolar lavage (BAL) cells on day 1. Cells in area O were gated as BAL mononuclear cells in the scatterplot of forward scatter (FS) and side scatter (SS) (**A**). CD4 T lymphocytes were identified through positive CD3 and CD4 (**B**). CD8 T lymphocytes were identified through positive CD3 and CD8 (**C**). Monocytes were identified through positive CD11b and CD14 (**D**). Expressions of human leukocyte antigen (HLA)-DR, interleukin (IL)-10, and IL-12 are represented in the histograms ((**E**–**G**) respectively). In this patient, HLA-DR, IL-10, and IL-12 were expressed in 79.79, 37.01, and 75.24% of monocytes. The mean fluorescence intensities of HLA-DR, IL-10 and IL12 were 92.28, 37.79, and 536.65, respectively.

BAL mononuclear cells (5 × 10^5^) were suspended in 50 μL of phosphate-buffered saline (PBS) and incubated in the dark for 15 min at room temperature with 10 μL antibodies of CD3_Alexa Fluor 700_, CD4_ECD_, CD8_APC_, CD11b_PC7_, CD14_APC-750_ and HLA-DR_FITC_. The CD4/CD8 T lymphocytes and mHLA-DR were detected by an eight-color flow cytofluorimeter (Beckman Coulter, Brea, CA, USA) (Figure 1).

BAL mononuclear cells (5 × 10^5^) were stimulated in vitro using 5 μg/mL phytohemaglutinin in 1 mL sterile RPMI1640 (Gibco, Grand Island, NY, USA) tissue culture medium with 5% heat-inactivated bovine serum, and 1 mM sodium pyruvate (Gibco, Grand Island, NY, USA). The cells were cultured for 4 h at 37 °C and 5% CO_2_. Then, the cells were suspended in 50 μL of PBS and incubated in the dark for 15 min at room temperature with 10 μL antibodies of CD11b_PC7_ and CD14_APC-750_. Then, the cells were washed and resuspended in 500 μL PBS with a permeabilizing solution (Beckman Coulter, Brea, CA, USA). The cells were washed and suspended in 50 μL PBS and incubated in the dark for 15 min at room temperature with 10 μL antibodies of IL-12_PC5.5_ and IL-10_Alexa Fluor 647_. Finally, the cells were washed and resuspended in 500 μL of PBS. The expressions of IL-10 and IL-12 in monocytes were detected using an eight-color flow cytofluorimeter (Beckman Coulter, Brea, CA, USA).

### 2.4. Statistical Analyses

We used the IBM SPSS Statistics, version 27.0.1 for Mac (IBM Inc., Armonk, NY, USA) to perform statistical analyses. Continuous variables were shown as means ± standard deviation. The differences between the two groups were analyzed using the Student’s *t*-test. Categorical variables were reported as numbers (percentages). The differences between categorical groups were compared by the Pearson’s chi-squared test or Fisher’s exact test. Differences in continuous variables in the same subjects were compared using a paired-samples *t*-test. Correlations between continuous variables were examined by the Pearson’s correlation test. *p* < 0.05 considered statistically significant.

## 3. Results

Table 1 shows the clinical characteristics of the survivors and non-survivors with ARDS. Twenty-five patients survived, two died within seven days, and one died within 8−28 days. The ventilator was successfully discontinued in two survivors within seven days. BAL was not performed on day 8 in these two survivors and two non-survivors. The APACHE II score and adverse event percentage of new arrhythmias in non-survivors were considerably higher than those in survivors. There were no differences in the percentages of gastrointestinal bleeding, acute kidney injury, shock, thrombocytopenia, jaundice, or bacteremia. Age, male percentage, history, partial pressure of oxygen/fraction of inspired oxygen ratio, and blood leukocytes in survivors were similar to those in non-survivors. On day eight, the APACHE II and SOFA scores in non-survivors were substantially higher than those in survivors.

### 3.1. Analysis of BAL Mononuclear Cells between Survivors and Non-Survivors

The percentages of CD4 and CD8 T lymphocytes and monocytes in BAL mononuclear cells between survivors and non-survivors were similar on days one and eight (Table 2). The expressions of HLA-DR, IL-10, and IL12 in monocytes were similar between survivors and non-survivors on days one and eight.

### 3.2. Analysis of BAL Mononuclear Cells between Day 1 and 8 in Survivors and Non-Survivors

**Figure 2:**
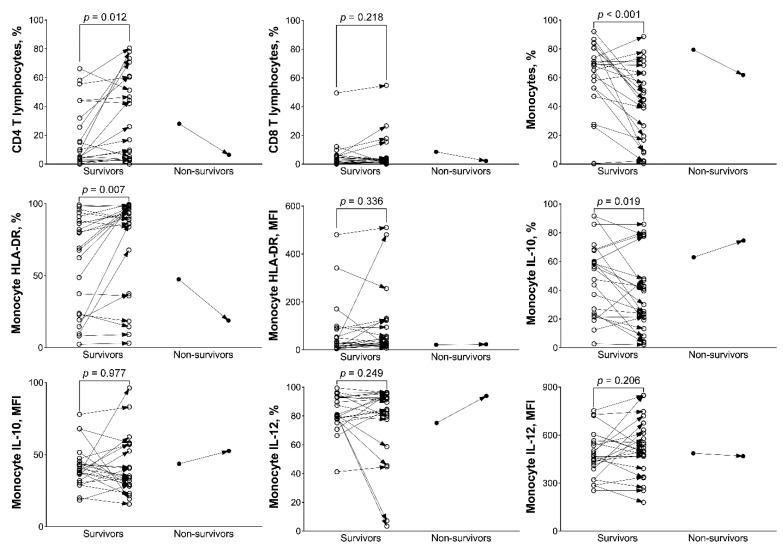
Percentages of bronchoalveolar lavage mononuclear cells, monocyte human leukocyte antigen (HLA)-DR expression, and monocyte intracellular interleukin (IL)-10/IL-12 expression between days 1 and 8 in survivors and non-survivors. Percentages of CD4 T lymphocytes and monocyte HLA-DR expression in percentage increased after 7 days in survivors. Monocyte HLA-DR expression in mean fluorescence intensity (MFI) did not change after 7 days in survivors. Monocyte percentage and monocyte IL-10 expression in percentage decreased after 7 days in survivors. Monocyte IL-10 expression in MFI did not change after 7 days in survivors.

The mHLA-DR and the percentage of CD4 T lymphocytes in BAL mononuclear cells of survivors were substantially increased after seven days (Figure 2). Monocyte IL-10 expression and the percentage of monocytes in BAL mononuclear cells of survivors were decreased after seven days. Monocyte IL-12 expression and the percentage of CD8 T lymphocytes in BAL mononuclear cells of survivors did not change after seven days. The difference between days one and eight in non-survivors was not compared due to availability of data from only one patient.

### 3.3. Correlation between BAL mHLA-DR and Severity Scores

**Figure 3:**
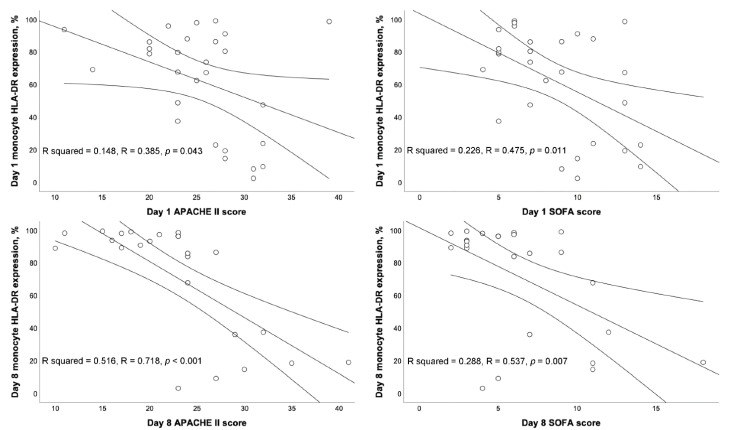
Scatterplots showing the correlation with linear regression lines (mean and 95% confidence interval) between monocyte human leukocyte antigen (HLA)-DR expression and Acute Physiology and Chronic Health Evaluation (APACHE) II score and Sequential organ failure assessment (SOFA) score on days 1 and 8. Pearson correlation coefficient (R) and the coefficient of determination (R squared) were calculated.

The mHLA-DR was negatively correlated with the APACHE II and SOFA scores on day 1 (R = −0.385, *p* = 0.043; R = −0.475, *p* = 0.011, respectively) (Figure 3). On day 8, mHLA-DR was also negatively correlated with APACHE II and SOFA scores (R = −0.718, *p* < 0.001; R = −0.537, *p* = 0.007, respectively).

## 4. Discussion

We first found that BAL mHLA-DR increased after seven days in survivors with pneumonia-related ARDS. The mHLA-DR was negatively correlated with APACHE II and SOFA scores. On day eight, the R squared between the mHLA-DR and APACHE II score was 0.516. This meant that 51.6% of mHLA-DR could be precisely predicted by the APACHE II score. In this study, only non-survivors with complete BAL on day eight showed a decreasing trend in mHLA-DR and an increasing trend in severity score. We can reasonably interpret that non-survivors might have decreased BAL mHLA-DR, as the trend of severity score in non-survivors was generally increased [14,16].

Many studies have reported that blood mHLA-DR was recovered in survivors with critical illness, compared with non-survivors [5,17,18,19]. Compared with septic patients without acute kidney injury (AKI), patients with AKI had significantly lower blood mHLA-DR [20]. In patients with ARDS, our study first demonstrated that survivors had an increased trend of HLA-DR expression in repeated measurements. Recovery of BAL mHLA-DR helps local organs increase cell-mediated immunity against pathogens and survival rate in patients with ARDS.

In patients with sepsis, no statistically significant correlation was found between blood mHLA-DR and APACHE II score [21]. In patients with acute pancreatitis, a strong inverse correlation between the mHLA-DR and APACHE II scores was observed [22]. This study is the first to demonstrate that BAL mHLA-DR was negatively correlated with APACHE II and SOFA scores on day one and eight. The degree of immune depression of mHLA-DR in the local infection area appears to be parallel to disease severity in patients with ARDS.

CD4 T lymphocytes facilitate the early clearance of bacteria by regulating neutrophil function, possibly through an interferon-dependent mechanism [23]. Patients with sepsis benefit from increased CD4 T lymphocyte count. Thus, it is easy to explain why the percentage of CD4 T lymphocytes in BAL fluid increased after seven days in survivors of ARDS, since patients with more CD4 T lymphocytes had a higher immune response to defend against pathogens and a higher possibility of survival. However, this point of view did not include regulatory T (T_reg_) cells because the ratio of T_reg_ cells to all CD4 cells in BAL of ARDS non-survivors was higher than that of survivors [24]. Our results were similar to those of a previous study that analyzed PBMCs and found that the percentage of CD4 T lymphocytes in survivors was significantly increased after six days [25].

The cause of the decreased trend in BAL monocyte percentage in survivors was unclear. One possible cause may be the increased percentage of CD4 T lymphocytes in BAL mononuclear cells. Due to the lack of the trend of BAL monocyte percentage in non-survivors, this statistically significant result may not be interpretated. In patients with septic shock, decreased monocyte IL-10 production positively correlated with mHLA-DR and tumor necrosis factor-α production [26]. Early plasma IL-10 level could predict the outcome in patients with ARDS receiving extracorporeal membrane oxygenation [27]. In this study, BAL monocyte IL-10 expression decreased after seven days in survivors. These suggested that the immune suppression by IL-10 from BAL monocytes decreased and the immune balance forwarded inflammation in the lungs of survivors. Many studies found that decreased IL-10 level in severe infection help patients survive [5,7,28,29,30]. Our results further demonstrated that decreased monocyte IL-10 expression in the local infectious area is beneficial for patients with ARDS.

This study has two limitations. First, the patients enrolled had an unexpected mortality rate lower than 20%, which resulted in insufficient case numbers in non-survivors to obtain BAL samples for experiments. Therefore, while the results of this study could not point to a definitive difference in the trend of BAL mHLA-DR and IL-10 expressions between survivors and non-survivors of ARDS, they did provide sufficient evidence to show the immunological trend in survivors. Further research is required in this area. Second, the number of patients was relatively low, resulting that strong conclusions cannot be derived.

## 5. Conclusions

This study showed a serial increase in HLA-DR expression and a decrease in IL-10 expression in BAL monocytes of survivors with pneumonia-related ARDS. Thus, an agent that can restore mHLA-DR and prevent IL-10 production in the infected lung might improve survival in patients with ARDS. As this study is the first to report these immune presentations, more large-scale studies are necessary to confirm our results.

## Figures and Tables

**Table 1:** Clinical characteristics of patients with ARDS (number, mean ± standard deviation).

	Survivors (n = 25)	Non-Survivors (n = 3)	All Patients (n = 28)
Age (years old)	71.8 ± 16.0	77.7 ± 18.0	72.5 ± 16.0
Male (%)	16 (64.0)	2 (66.7)	18 (64.3)
APACHE II score	24.7 ± 5.1	32.3 ± 6.5 *	25.4 ± 5.7
SOFA score	8.4 ± 3.1	11.0 ± 3.5	8.6 ± 3.2
History (%)			
COPD	4 (16.0)	1 (33.3)	5 (17.9)
Heart failure	1 (4.0)	0 (0.0)	1 (3.6)
Hypertension	12 (48.0)	3 (100.0)	15 (53.6)
Diabetes mellitus	10 (40.0)	1 (33.3)	11 (39.3)
Previous CVA	6 (24.0)	1 (33.3)	7 (25.0)
End stage renal disease	0 (0.0)	0 (0.0)	0 (0.0)
Liver cirrhosis	1 (4.0)	0 (0.0)	1 (3.6)
Active malignancy	0 (0.0)	1 (33.3)	1 (3.6)
Adverse event			
New arrhythmia	1 (4.0)	2 (66.7) *	3 (10.7)
Gastrointestinal bleeding	6 (24.0)	0 (0.0)	6 (21.4)
Acute kidney injury	8 (32.0)	2 (66.7)	10 (35.7)
Shock	6 (24.0)	2 (66.7)	8 (28.6)
Thrombocytopenia	6 (24.0)	2 (66.7)	8 (28.6)
Jaundice	3 (12.0)	0 (0.0)	3 (10.7)
Bacteremia	4 (16.0)	1 (33.3)	5 (17.9)
PaO_2_/FiO_2_ ratio (mm Hg)	151.9 ± 64.3	177.0 ± 49.8	154.6 ± 62.6
Blood leukocytes			
WBC/μL	11,480.0 ± 8683.7	11,466.7 ± 1601.0	11,478.6 ± 8198.7
Neutrophils, %	84.6 ± 9.2	89.7 ± 5.2	85.2 ± 9.0
Lymphocytes, %	10.3 ± 8.1	7.0 ± 4.3	9.9 ± 7.8
Monocytes, %	4.0 ± 2.0	3.0 ± 2.1	3.9 ± 2.0
Day 8	(n = 23)	(n = 1)	(n = 24)
APACHE II score	22.1 ± 6.3	41.0 *	22.9 ± 7.3
SOFA score	6.0 ± 3.2	18.0 *	6.5 ± 4.0

Abbreviations: ARDS, acute respiratory distress syndrome; APACHE, Acute Physiology and Chronic Health Evaluation; SOFA, sequential organ failure assessment; COPD, chronic obstructive pulmonary disease; CVA, cerebral vascular accident; PaO_2_, partial pressure of oxygen; FiO_2_, fraction of inspired oxygen; WBC, white blood cell. * *p* < 0.05 compared with survivors using *t*-test.

**Table 2:** BAL mononuclear cells in patients with ARDS (mean ± standard deviation).

	Survivors	Non-Survivors	*p* Value
Day 1	(n = 25)	(n = 3)	
CD4 T lymphocytes, %	16.2 ± 21.0	27.1 ± 8.4	0.389
CD8 T lymphocytes, %	4.8 ± 9.9	7.1 ± 1.3	0.685
Monocytes, %	59.3 ± 27.0	59.9 ± 18.3	0.967
Monocyte HLA-DR expression, %	60.8 ± 33.0	71.2 ± 25.8	0.605
Monocyte HLA-DR expression, MFI	69.1 ± 112.0	150.6 ± 228.2	0.295
Monocyte IL-10 expression, %	47.7 ± 24.7	42.8 ± 21.3	0.742
Monocyte IL-10 expression, MFI	41.7 ± 13.7	43.0 ± 3.2	0.870
Monocyte IL-12 expression, %	82.3 ± 13.0	84.7 ± 8.7	0.762
Monocyte IL-12 expression, MFI	479.7 ± 137.7	621.8 ± 146.9	0.105
Day 8	(n = 23)	(n = 1)	
CD4 T lymphocytes, %	30.1 ± 29.6	6.4	0.443
CD8 T lymphocytes, %	6.7 ± 12.3	2.2	0.722
Monocytes, %	41.6 ± 26.9	61.9	0.469
Monocyte HLA-DR expression, %	73.2 ± 33.8	18.9	0.131
Monocyte HLA-DR expression, MFI	93.2 ± 139.7	23.2	0.629
Monocyte IL-10 expression, %	37.3 ± 26.9	74.6	0.188
Monocyte IL-10 expression, MFI	41.4 ± 20.2	52.7	0.590
Monocyte IL-12 expression, %	76.7 ± 27.0	93.9	0.540
Monocyte IL-12 expression, MFI	520.1 ± 177.9	469.3	0.783

Abbreviations: BAL, bronchoalveolar lavage; ARDS, acute respiratory distress syndrome; HLA, human leukocyte antigen; MFI, mean fluorescence intensity; IL = interleukin.

## Data Availability

The datasets generated for this study can be obtained on request from the corresponding author.
